# Propofol in Perioperative Management of Head and Neck Cancer: A Narrative Review of Molecular Mechanisms and Clinical Implications

**DOI:** 10.3390/cimb48070708

**Published:** 2026-07-11

**Authors:** Yu Sun, Po-Chih Hsu, Chung-Che Tsai, Tsui-Chin Peng, Chan-Yen Kuo

**Affiliations:** 1 Department of Dentistry, Taipei Tzuchi Hospital, The Buddhist Tzu Chi Medical Foundation, New Taipei City 23142, Taiwan; fiona0113@tzuchi.com.tw (Y.S.); pchsu@gms.tcu.edu.tw (P.-C.H.); or tch43094@tzuchi.com.tw (C.-C.T.); 2 Institute of Oral Medicine and Materials, College of Medicine, Tzu Chi University, Hualien 970004, Taiwan; 3 Department of Anesthesia, Taipei Tzuchi Hospital, The Buddhist Tzu Chi Medical Foundation, New Taipei City 23142, Taiwan; christineptc1110@gmail.com

**Keywords:** propofol, head and neck cancer, oral squamous cell carcinoma, perioperative oncology, tumor microenvironment, PI3K/AKT/mTOR, epithelial–mesenchymal transition, immune modulation, total intravenous anesthesia, cancer progression

## Abstract

The potential impact of propofol on head and neck cancer (HNC) progression and clinical outcomes, in relation to both direct effects on tumor biology and indirect effects mediated by perioperative immune and inflammatory responses, remains controversial. Most mechanistic evidence currently available for HNC originates from studies of oral squamous cell carcinoma. Accordingly, mechanistic findings are interpreted primarily as OSCC-derived evidence and should not be generalized to all HNC subsites without further validation. To address this, we critically examined propofol’s potential role as a perioperative anesthetic modulator of tumor biology and host responses. Preclinical studies have demonstrated that propofol suppresses proliferation, induces apoptosis, inhibits angiogenesis, and enhances 5-fluorouracil chemosensitivity. Conversely, other studies report enhanced epithelial–mesenchymal transition, migration, and invasion through SNAI1 upregulation, highlighting the context-dependent nature of propofol-mediated effects. The generalizability of these molecular observations to other HNC subsites, including laryngeal, hypopharyngeal, and oropharyngeal cancers, remains unclear. Clinically, propofol-based total intravenous anesthesia has been associated with reduced postoperative pulmonary complications and improved perioperative recovery. However, currently available HNC-specific studies have not demonstrated a consistent improvement in overall or recurrence-free survival. These discrepancies likely reflect tumor heterogeneity, variations in experimental design, perioperative confounding factors, and differences in host immune and inflammatory responses. This narrative review was based on literature identified through PubMed, Scopus, and Google Scholar, and integrates current mechanistic, immunological, and clinical evidence regarding propofol exposure during cancer surgery. Current evidence supports the perioperative benefits of propofol-based anesthesia; however, its long-term oncologic significance in HNC remains inconclusive and warrants further prospective, mechanism-driven, and biomarker-guided investigations.

## 1. Introduction

Head and neck cancer (HNC), most represented by head and neck squamous cell carcinoma (HNSCC), remains a major global health burden despite advances in surgery, radiotherapy, and systemic therapies. Throughout this review, the term HNC is used when discussing the broader clinical disease spectrum, whereas HNSCC is used when referring specifically to squamous cell carcinoma-related evidence [1,2]. Therefore, increasing attention has been focused on perioperative factors that may influence tumor progression, including anesthetic techniques [3].

Propofol is not a therapeutic agent for HNC but rather one of the most commonly used intravenous anesthetics during cancer surgery. Interest in propofol has emerged from accumulating evidence suggesting that perioperative anesthetic exposure may influence tumor biology, host immune responses, and postoperative oncological outcomes. Therefore, the potential role of propofol in perioperative oncology should be interpreted in the context of anesthetic management rather than in direct anti-cancer therapy. Propofol (2,6-diisopropylphenol) is a short-acting intravenous anesthetic widely used for the induction and maintenance of anesthesia [4]. In addition to its sedative effects, propofol may influence tumor biology by modulating oncogenic signaling pathways, metabolic reprogramming, and anti-tumor immune responses [5]. Compared with inhalational anesthesia (INH) agents such as sevoflurane, propofol-based total intravenous anesthesia (TIVA) has been associated with perioperative advantages, including less emergence agitation and lower airway reactivity. However, current evidence shows no clear difference in early postoperative recovery quality or oncologic outcomes [6]. Although some retrospective studies and meta-analyses have suggested a potential survival benefit of propofol-based TIVA, these findings are inconsistent and constrained by study design. Notably, HNC-specific studies have not demonstrated significant improvements in overall survival (OS) or recurrence-free survival (RFS), underscoring the context-dependent and unresolved nature of propofol’s oncologic relevance in HNC [7]. These inconsistencies highlight the need for a comprehensive and critical evaluation of both the mechanistic and clinical evidence. The oncologic effects of anesthetic agents are increasingly recognized as multifactorial and may differ substantially according to tumor subtype, host immune status, perioperative inflammatory responses, and patient-related clinical factors [8,9,10,11]. In HNC, substantial biological heterogeneity exists among the oral cavity, laryngeal, hypopharyngeal, and HPV-associated oropharyngeal cancers, which may contribute to differential responses to perioperative anesthetic exposure [12,13,14,15,16]. In addition, perioperative immune modulation involving inflammatory cytokines, natural killer (NK) cell activity, and T-cell function has emerged as a potentially important determinant of tumor recurrence and metastasis [11,17,18,19]. These observations highlight the need to evaluate propofol not only as an anesthetic agent but also as a potential modulator of the tumor microenvironment and perioperative host responses.

In this narrative review, we specifically address three questions regarding (i) OSCC molecular mechanisms, (ii) HNC-specific clinical outcomes, and (iii) perioperative immune modulation, with the aim of identifying the major evidence gaps that currently limit clinical translation. Rather than evaluating propofol as a cancer treatment, we focused on its potential influence as a perioperative anesthetic agent that may affect tumor biology, immune regulation, and clinical outcomes in patients undergoing cancer surgery. Particular emphasis is placed on explaining the apparent discrepancies across studies and identifying the future research priorities needed to translate these findings into clinical practice. Although several cited studies are narrative reviews or meta-analyses, these references were included only when they provided direct mechanistic, immunological, or translational insights relevant to propofol, perioperative tumor biology, or HNC. Studies without a clear biological or clinical connection to these topics were excluded. Furthermore, HNC-specific evidence was preferentially cited whenever available, whereas evidence derived from broader perioperative oncology or mixed cancer populations is explicitly identified throughout this review to avoid inappropriate extrapolation [4,5,7,8,9,14,20,21,22,23,24,25]. Nevertheless, the limited availability of adequately powered randomized controlled trials (RCTs), particularly HNC-specific prospective studies, remains a major limitation of current evidence and highlights the need for future mechanism-driven clinical investigations. Given this limited availability of adequately powered RCTs in HNC-specific populations, a purely evidence-synthesis approach may not fully capture the biological complexity and translational relevance of propofol in cancer surgery. Therefore, this review is designed not only to summarize current clinical findings but also to integrate emerging mechanistic evidence with perioperative immunological concepts and translational perspectives. By bridging molecular mechanisms with clinical observations, we aim to provide a more comprehensive framework for understanding the context-dependent role of propofol in perioperative oncology and identify critical gaps that remain unresolved in current clinical research.

Previous reviews have primarily focused on either the molecular actions of propofol across multiple cancer types or the relationship between anesthetic techniques and oncologic outcomes in heterogeneous cancer populations [4,5,7,8,10,11,14,20,26]. However, none has comprehensively integrated OSCC-derived mechanistic evidence with HNC-specific clinical studies while systematically distinguishing direct tumor-intrinsic mechanisms from indirect perioperative host-mediated immune responses. Consequently, the translational significance of current evidence remains difficult to interpret, particularly for perioperative decision-making in HNC [8,9,10,11,26].

Because the available evidence originates from different study designs and populations, Table 1 summarizes the evidence hierarchy and distinguishes HNC-specific clinical evidence from broader perioperative oncology literature. This classification provides the framework used throughout the review to interpret the strength and translational relevance of the available evidence.

### 1.1. Literature Search and Evidence Selection

This narrative review was based on a structured literature search conducted in PubMed, Scopus, and Google Scholar. The final search was performed on 30 June 2026, and studies published between January 2000 and June 2026 were considered. Representative search terms included (“propofol” OR “total intravenous anesthesia” OR “TIVA”) AND (“head and neck cancer” OR “head and neck squamous cell carcinoma” OR “HNSCC” OR “oral squamous cell carcinoma” OR “OSCC”) AND (“perioperative oncology” OR “immune modulation” OR “tumor microenvironment” OR “cancer outcomes”).

Eligible publications included original experimental studies, randomized controlled trials, prospective and retrospective clinical studies, systematic reviews, meta-analyses, and influential narrative reviews published in English. Conference abstracts, editorials, letters without original data, duplicate publications, and studies unrelated to propofol or perioperative oncology were excluded.

Titles and abstracts were initially screened for relevance, followed by full-text evaluation of potentially eligible articles. Studies were selected according to their relevance to the predefined review questions, methodological quality, and contribution to understanding molecular mechanisms, perioperative immune modulation, or HNC-specific clinical outcomes. Data extraction focuses on study design, cancer type, experimental model or patient population, propofol exposure, principal molecular pathways, immune-related findings, perioperative outcomes, and oncologic outcomes.

Because this work was designed as a narrative review rather than a systematic review, a formal risk-of-bias assessment and PRISMA-guided study selection were not performed. Nevertheless, evidence was critically appraised and prioritized according to an evidence hierarchy, with randomized controlled trials, systematic reviews, and meta-analyses considered the highest level of clinical evidence, followed by prospective and retrospective clinical studies, while mechanistic and preclinical studies were primarily used to support biological interpretation and identify knowledge gaps.

Distinguishing between HNC-specific evidence and observations derived from broader perioperative oncology studies is a central principle of this review. Most mechanistic studies have been conducted using OSCC models, whereas evidence regarding perioperative immune modulation, inflammatory cytokine regulation, and potential survival benefits is largely derived from mixed cancer cohorts or non-HNC malignancies. Therefore, the extrapolation of these findings to HNC should be interpreted with caution. Accordingly, HNC-specific evidence and broader perioperative oncology evidence are presented separately throughout the review to facilitate interpretation of the strength, applicability, and translational relevance of the available data.

References from broader perioperative oncology or non-HNC studies were included only when they provided mechanistic, immunological, or translational insights directly relevant to propofol, perioperative host responses, or HNC biology. Studies lacking a clear connection to these topics were excluded.

### 1.2. Rationale and Review Questions

Although increasing evidence suggests that propofol may influence cancer biology during the perioperative period, its clinical significance in head and neck cancer (HNC) remains uncertain because available evidence is fragmented across mechanistic studies, perioperative immunology, and heterogeneous clinical investigations. Most molecular data originate from oral squamous cell carcinoma (OSCC), whereas clinical outcome studies often include mixed HNC populations or broader cancer cohorts. Consequently, the translational relevance of current findings remains difficult to interpret.

To address these unresolved issues, this narrative review was designed to answer three specific questions:

(1) What molecular mechanisms have been identified through which propofol modulates tumor biology in OSCC, and how robust is the current mechanistic evidence?

(2) What HNC-specific clinical evidence exists regarding the effects of propofol-based anesthesia on perioperative outcomes and long-term oncologic outcomes?

(3) How might perioperative immune modulation and host responses explain the discrepancy between experimental findings and clinical observations, and what evidence gaps should be prioritized in future research?

By integrating mechanistic, immunological, and clinical evidence within a single translational framework, this review aims to clarify current controversies, distinguish HNC-specific evidence from broader perioperative oncology literature, and identify key directions for future mechanism-driven and biomarker-guided investigations.

Evidence was prioritized according to the review methodology described in Section 1.1, with randomized controlled trials, systematic reviews, and meta-analyses considered the highest level of clinical evidence, followed by observational clinical studies and mechanistic preclinical investigations.

## 2. HNC-Specific Clinical Evidence

### 2.1. Survival Outcomes and Oncologic Prognosis

Current evidence regarding the impact of propofol-based total intravenous anesthesia (TIVA) on overall survival (OS), recurrence-free survival (RFS), and other long-term oncologic outcomes in patients with HNC remains inconclusive. Although several retrospective studies and meta-analyses have suggested potential survival associations, currently available HNC-specific studies have not demonstrated consistent improvements in OS or RFS. Although some retrospective studies and broader meta-analyses on cancer surgery have reported associations between propofol-based anesthesia and improved OS or RFS, available HNC-specific studies have not demonstrated consistent survival benefits [20,36]. For example, Miao et al. suggested that propofol-based TIVA and sevoflurane-based INH are acceptable options for oral cancer surgery without adversely affecting long-term oncological outcomes [36]. In contrast, Tang et al. reported that propofol-based anesthesia may be associated with improved OS and RFS in selected cancer populations, particularly in single-center studies [20]. To facilitate interpretation of the available literature, Table 2 summarizes the highest-level clinical evidence currently available regarding propofol exposure and cancer outcomes. Although several systematic reviews, meta-analyses, and retrospective cohort studies have been published, HNC-specific randomized clinical data remain limited. To date, no adequately powered prospective clinical trial has demonstrated consistent improvements in OS or RFS associated with propofol-based anesthesia in patients with HNC. Unless otherwise specified, the clinical studies discussed in this section represent the highest level of currently available evidence directly relevant to propofol-based anesthesia and perioperative oncology. Findings derived from mixed cancer populations are interpreted only as supportive evidence and are not considered equivalent to HNC-specific clinical evidence.

A major limitation of current literature is the predominance of retrospective observational studies, which are inherently susceptible to selection bias and residual confounding [39,40]. Moreover, prospective randomized data remain limited, and findings from retrospective analyses have not been consistently confirmed in controlled trials [12,13]. Additionally, substantial heterogeneity exists across studies regarding tumor type, stage, perioperative variables, and adjuvant therapies. Oral cavity, laryngeal, and mixed HNC cohorts are often analyzed together despite marked differences in biology and clinical management, further complicating interpretation [14,15,16]. For instance, a retrospective study demonstrated that propofol infusion did not confer a survival benefit to patients with HNSCC [15]. Collectively, these limitations underscore the need for well-designed and adequately powered prospective studies with standardized stratification and perioperative protocols to clarify the prognostic relevance of anesthetic exposure.

Anesthetic exposure is rarely an isolated variable. Perioperative outcomes are influenced by multiple interacting factors, including opioid use, intraoperative transfusion, surgical stress, and systemic inflammatory responses, all of which may influence immune function and tumor biology. These factors may have confounded the observed associations between propofol exposure and oncologic outcomes [8,9,23]. Moreover, their impact is often further magnified in retrospective studies.

### 2.2. Perioperative Outcomes

Unlike long-term oncological outcomes, the perioperative benefits of propofol appear to be more consistent. Several studies have reported reduced postoperative pulmonary complications (PPCs), shorter hospital stays, and improved recovery profiles in patients receiving TIVA, particularly in those undergoing prolonged surgeries with microvascular reconstruction [33,34]. A randomized clinical trial evaluated the effects of TIVA versus INH on PPCs in patients undergoing microvascular reconstruction for HNC. The incidence of PPCs was significantly lower in the TIVA group than in the INH group. These findings suggest that propofol-based TIVA may reduce the risk of postoperative pulmonary complications in this specific surgical setting; however, these perioperative benefits should not be interpreted as evidence of improved long-term oncologic outcomes or generalized to all HNC populations [33]. Lee et al. examined patients undergoing anatomical pulmonary resection and compared the outcomes of TIVA and INH. Their results showed that TIVA was associated with a lower risk of PPCs, reinforcing the role of propofol in preserving respiratory function and improving perioperative recovery in thoracic surgical settings [34]. Another nationwide cohort study by Oh et al. focused on patients undergoing spinal surgery, and reported that propofol-based TIVA was associated with decreased in-hospital mortality and fewer postoperative complications than INH. These studies provide supportive evidence that the perioperative benefits of propofol-based TIVA may extend across different surgical populations; however, they should not be interpreted as HNC-specific evidence because tumor biology, operative procedures, and perioperative risk profiles differ substantially between surgical populations [35]. In summary, propofol-based TIVA demonstrates relatively consistent perioperative benefits, including reduced postoperative pulmonary complications and improved postoperative recovery. However, these perioperative advantages have not translated into consistent improvements in OS, RFS, or other long-term survival outcomes in HNC-specific studies.

## 3. OSCC-Derived Mechanistic Evidence

In recent years, accumulating evidence suggests that propofol may exert both anti-tumor and pro-tumor effects through direct action on tumor cells, including the modulation of cell behavior and regulation of drug resistance [21,22,31,32]. These contradictory effects of propofol on carcinogenesis may depend on the tumor type or specific conditions [21]. Li et al. reported that clinically relevant concentrations of propofol enhance cell migration and invasion in OSCC cells by upregulating the Snail family transcriptional repressor 1 (SNAI1), suggesting that propofol may be unsuitable for anesthesia management in patients with OSCC [41]. In contrast, propofol may inhibit OSCC progression by modulating the circ_0005623/miR-195-5p/HOXB7 axis [27]. Furthermore, propofol suppresses OSCC proliferation, invasion, migration, and angiogenesis by modulating the circ_0008898-mediated signaling pathway [28].

### 3.1. Preclinical Molecular Mechanisms of Propofol in Oral Squamous Cell Carcinoma

Previous studies have shown that propofol reduces OSCC cell viability and promotes apoptosis. Additionally, propofol reduces the expression and secretion of amphiregulin (AREG), a growth factor associated with poor prognosis and drug resistance in OSCC. Notably, propofol ameliorates 5-fluorouracil resistance in OSCC cells by downregulating AREG expression [29]. It has also been reported to exert anti-tumor effects in OSCC by inducing apoptosis and inhibiting cell growth through activation of the FoxO1-GAS5-miR-1297-GSK3β axis [38]. A comprehensive review highlighted that the PI3K/AKT/mTOR pathway is frequently altered in OSCC and influences key cellular and metabolic processes. By examining the upstream activators and downstream effectors, the authors suggested that targeting components of this pathway could offer promising therapeutic strategies for OSCC treatment [30].

**Figure 1:**
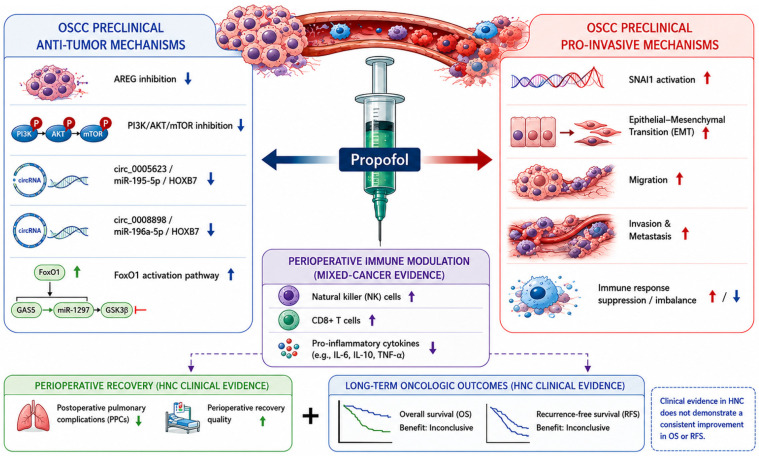
**Evidence-anchored overview of the reported anti-tumor and pro-invasive effects of propofol in head and neck cancer (HNC).** The (**left panel**) summarizes OSCC-derived preclinical evidence indicating that propofol suppresses tumor progression through inhibition of the AREG, PI3K/AKT/mTOR, and non-coding RNA–associated signaling pathways, while promoting apoptosis and enhancing chemosensitivity. The (**right panel**) summarizes OSCC-derived preclinical studies reporting activation of SNAI1, epithelial–mesenchymal transition (EMT), migration, invasion, metastasis, and immune suppression under specific experimental conditions. The (**lower panel**) integrates mixed-cancer perioperative evidence demonstrating potential modulation of perioperative immune responses and HNC-specific clinical evidence showing improved perioperative recovery, whereas current clinical studies do not consistently demonstrate improvements in overall survival (OS) or recurrence-free survival (RFS). Colored symbols indicate the evidence source: blue, OSCC preclinical evidence; orange, mixed-cancer perioperative clinical evidence; green, HNC-specific clinical evidence; dashed arrows, hypothetical or incompletely validated interactions. Reference numbers correspond to the key studies summarized in Table 2, Table 3 and Table 4. This figure was conceptually designed by the authors based on evidence synthesized from preclinical OSCC studies, mixed-cancer perioperative oncology studies, and HNC-specific clinical investigations summarized in this review. The illustration is intended as an evidence-anchored conceptual framework and does not imply equivalent levels of evidence or established causal relationships among the depicted mechanisms. Most molecular pathways shown are derived from experimental OSCC models and should therefore be interpreted as biological plausibility rather than clinically validated mechanisms in patients with HNC. Dashed lines indicate hypothetical relationships; ↑ Upregulation; ↓ Downregulation. This figure was generated with the assistance of ChatGPT (OpenAI, GPT-5.5) and subsequently curated by the authors to ensure accuracy in biological representation, terminology, and conceptual integration.

**Figure 2:**
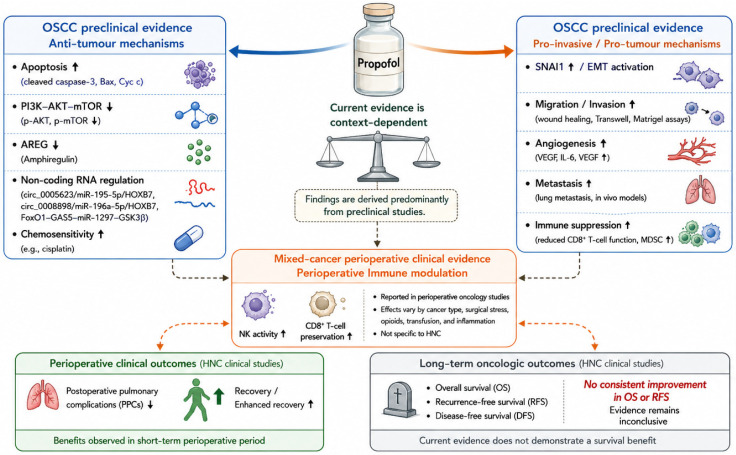
**Evidence-anchored overview of the context-dependent effects of propofol on tumor biology, perioperative immune modulation, and clinical outcomes in head and neck cancer.** Propofol has been reported to exert context-dependent effects on tumor biology through modulation of tumor-intrinsic signaling pathways and perioperative host responses. **Preclinical evidence derived predominantly from oral squamous cell carcinoma (OSCC) models** indicates potential anti-tumor effects, including induction of apoptosis and inhibition of PI3K–AKT–mTOR signaling, amphiregulin (AREG) expression, and non-coding RNA-associated regulatory pathways. In contrast, other OSCC experimental studies have reported activation of epithelial–mesenchymal transition (EMT), SNAI1 signaling, angiogenesis, migration, and invasion under specific experimental conditions. These divergent findings likely reflect differences in experimental models, propofol exposure conditions, and tumor microenvironmental context. Evidence for perioperative immune modulation is derived primarily from mixed-cancer perioperative clinical studies, which suggest preservation of natural killer (NK) cell activity, maintenance of CD8^+^ T-cell function, and attenuation of perioperative inflammatory responses. Because these observations originate largely from non-HNC populations, they should be interpreted as supportive perioperative evidence rather than HNC-specific clinical evidence. ↑ Upregulation; ↓ Downregulation. This figure was generated with the assistance of ChatGPT (OpenAI, GPT-5.5) and subsequently curated by the authors to ensure accuracy in biological representation, terminology, and conceptual integration.

Collectively, these findings highlight the complex and context-dependent roles of propofol in OSCC (Table 3). Importantly, the molecular mechanisms summarized in this section are derived predominantly from experimental cell-line models and therefore represent preclinical mechanistic evidence rather than clinically validated therapeutic mechanisms. Their translational relevance to patients with HNC requires further prospective validation. Collectively, these findings indicate that propofol exerts context-dependent biological effects in OSCC. However, because the available evidence is derived predominantly from experimental models, these molecular observations should be regarded as hypothesis-generating rather than clinically validated mechanisms. Translational takeaway: Current mechanistic evidence supports biological plausibility but requires validation in clinically relevant HNC models and prospective clinical studies before influencing perioperative decision-making. The context-dependent dual effects of propofol on OSCC are summarized in Figure 1. To further integrate the interactions between tumor-intrinsic signaling pathways, perioperative immunity, and clinical outcomes, a broader conceptual framework summarizing the context-dependent effects of propofol on HNC is presented in Figure 2.

### 3.2. Molecular Mechanisms Underlying the Dual Effects of Propofol

Although most experimental studies suggest that propofol exerts anti-tumor effects in OSCC, several investigations have reported pro-invasive or pro-migratory responses. Rather than representing inherently contradictory findings, these divergent observations are likely attributable to differences in experimental models, propofol exposure conditions, molecular endpoints, pathway validation strategies, and study design. To facilitate comparison across studies, the major methodological characteristics and mechanistic evidence of the primary OSCC investigations are summarized in Table 4.

**Table 4:** Mechanistic comparison of primary OSCC studies investigating the context-dependent effects of propofol.

Cell Line/Model	Propofol Exposure	Main Endpoint	Molecular Pathway	Validation	Rescue Experiment	Biological Direction	Translational Relevance/Limitation	References
SCC-9, CAL27	5, 10, 20 μM, 48 h	Migration ↑, invasion ↑	SNAI1/EMT	Wound-healing assay, Transwell assay, qPCR, Western blot	SNAI1 siRNA knockdown	Pro-invasive	Limited	[41]
SCC-9	20 μg/mL, 48 h	Proliferation ↓	circ_0005623	qPCR	miR-195-5p mimic	Anti-tumor	Moderate	[27]
SAS	5 μg/mL, 24 h	5-FU sensitivity ↑	AREG	WB, ELISA	Recombinant AREG	Anti-tumor	Moderate	[29]
CAL27	20 μg/mL, 48 h	Apoptosis ↑	GAS5	qPCR	siGAS5	Anti-tumor	Moderate	[38]
OSCC	8 μg/mL, 24 h	Angiogenesis ↓	circ_0008898	qPCR	Knockdown	Anti-tumor	Moderate	[28]

**Abbreviations:** EMT, epithelial–mesenchymal transition; SNAI1, snail family transcriptional repressor 1; qPCR, quantitative polymerase chain reaction; WB, Western blot; ELISA, enzyme-linked immunosorbent assay; siRNA, small interfering RNA; OSCC, oral squamous cell carcinoma; AREG, amphiregulin; circRNA, circular RNA; miRNA, microRNA; 5-FU, 5-fluorouracil; TIVA, total intravenous anesthesia. Clinical plausibility was qualitatively assessed based on the relevance of the experimental propofol exposure conditions to clinically achievable perioperative concentrations and the overall translational applicability of the study design. Clinically relevant plasma concentrations during propofol-based TIVA are generally estimated to range between approximately 2–6 μg/mL (11–33 μM). Exposure conditions substantially exceeding this range or extending beyond typical perioperative durations should be interpreted cautiously when considering clinical translation. ↑ Upregulation; ↓ Downregulation.

Clinical outcome data are derived predominantly from HNC-specific clinical studies. Current evidence consistently supports improved short-term perioperative outcomes, including reduced postoperative pulmonary complications (PPCs) and enhanced postoperative recovery. In contrast, available clinical studies have not consistently demonstrated improvements in long-term oncologic outcomes, including overall survival (OS) or recurrence-free survival (RFS). Evidence presented in this figure was selected according to the literature selection strategy described in Section 1.1, prioritizing HNC-specific studies whenever available. Non-HNC studies were included only when they provided direct mechanistic or perioperative biological evidence relevant to HNC. Tumor-intrinsic molecular mechanisms are derived predominantly from preclinical OSCC studies, perioperative immune modulation is based mainly on mixed-cancer perioperative oncology studies, and clinical outcome components represent HNC-specific clinical evidence where available. Solid arrows indicate evidence-supported relationships, whereas dashed arrows denote hypothetical, indirect, or incompletely validated interactions. Figure created with the assistance of ChatGPT (OpenAI) and subsequently reviewed, revised, and scientifically validated by the authors. This figure is intended as an evidence-anchored conceptual synthesis and should not be interpreted as implying equivalent levels of evidence among preclinical OSCC studies, mixed-cancer perioperative clinical studies, and HNC-specific clinical studies.

## 4. Evidence from Mixed Cancer Populations and Perioperative Immunology

Beyond its direct effects on cancer cells, propofol has been reported to modulate perioperative immune and inflammatory responses, including effects on cytotoxic T cells, NK-cell activity, and cytokine production, which may have implications for the tumor microenvironment [17]. Recent evidence suggests that the perioperative period is a biologically vulnerable phase in cancer progression. Surgical stress and associated factors, such as inflammation, pain, anemia, and perioperative interventions, can impair both innate and adaptive immune responses, thereby weakening tumor immune surveillance [10,11]. Propofol has been shown to mitigate the adverse effects of surgical stress on immune function and exert immunomodulatory effects, potentially contributing to improved perioperative outcomes [18].

Emerging evidence suggests that propofol may influence perioperative cytokine profiles by attenuating pro-inflammatory mediators such as interleukin (IL)-6, tumor necrosis factor-alpha (TNF-α), and cyclooxygenase-2 (COX-2)-associated signaling pathways while partially preserving anti-tumor immune activity [10,11,18,19,24,25]. These immunomodulatory effects may contribute to reduced perioperative immune dysfunction and improved recovery after major cancer surgery. However, the magnitude and clinical relevance of these effects likely vary according to tumor subtype, baseline inflammatory status, nutritional condition, smoking history, and patient comorbidities, such as diabetes mellitus, obesity, cardiovascular disease, or chronic pulmonary disease, all of which may independently alter perioperative immune and inflammatory responses [8,9,10,11,17].

Perioperative vascular and endothelial alterations may also contribute to tumor progression and immune dysregulation. Endothelial dysfunction, vascular remodeling, inflammatory activation, and abnormal microvascular responses may influence tissue hypoxia, immune cell trafficking, angiogenesis, and tumor dissemination during the perioperative period [10,11,18]. These findings support the broader concept that perioperative changes in the vascular microenvironment may interact with immune modulation and tumor biology in a context-dependent manner. Propofol also attenuates perioperative immunosuppression and supports the preservation of anti-tumor immune functions, particularly in the context of surgical stress [19]. Overall, propofol may help to preserve immune function and reduce perioperative immunosuppression during the critical window of cancer progression. However, its impact on long-term oncological outcomes remains uncertain and requires further prospective validation.

## 5. Limitations and Future Research Priorities

Current clinical evidence regarding the oncological effect of propofol on HNC remains limited and should be interpreted with caution. First, most available studies are retrospective observational analyses, which are inherently susceptible to selection bias, residual confounding, and institutional practice variation [15,20,36]. Important perioperative factors, including opioid exposure, blood transfusion, surgical complexity, postoperative complications, and adjuvant treatment strategies, may independently influence oncological outcomes and are difficult to control in retrospective studies [8,9,11,17,23].

Second, most published studies evaluated heterogeneous cancer populations rather than HNC-specific cohorts. Although several systematic reviews and meta-analyses have suggested potential survival advantages associated with propofol-based anesthesia, these findings are largely derived from mixed cancer populations and have yielded conflicting conclusions [4,7,20]. In contrast, currently available HNC-specific studies have generally failed to demonstrate a consistent improvement in OS or RFS [15,36].

Third, substantial biological heterogeneity exists within HNC itself. Oral cavity, oropharyngeal, hypopharyngeal, and laryngeal cancers differ considerably in their molecular characteristics, HPV status, immune landscapes, treatment approaches, and clinical behavior [12,13,14,15,16]. These differences may influence susceptibility to anesthetic-related biological effects but are rarely accounted for in currently available clinical studies.

Fourth, mechanistic evidence remains predominantly derived from OSCC cell lines and experimental systems [5,21,22,27,28,29,30,31,32]. During propofol-based total intravenous anesthesia (TIVA), clinically relevant plasma concentrations are generally maintained at approximately 2–6 μg/mL (approximately 11–33 μM). However, several experimental studies have used substantially higher propofol concentrations (e.g., ≥50 μM) and/or prolonged exposure durations (48–72 h), which exceed the pharmacokinetic conditions typically encountered during routine surgery. These methodological differences may substantially influence the observed biological responses and should be considered when interpreting the translational significance of current preclinical findings [21,22]. Therefore, the translational significance of these findings remains unclear.

Fifth, the perioperative tumor microenvironment is influenced by numerous interacting factors, including surgical stress, systemic inflammation, immune suppression, endothelial dysfunction, and metabolic alterations [8,9,10,11,17,18,19,24,37,42]. Distinguishing the independent contribution of anesthetic exposure from other perioperative determinants of cancer progression and recurrence remains challenging.

Finally, adequately powered prospective randomized clinical trials specifically designed for HNC remain scarce. Consequently, current evidence is insufficient to establish a causal relationship between perioperative propofol exposure and long-term oncologic outcomes [4,7,20,37]. Collectively, these methodological limitations substantially restrict the clinical translation of the current evidence.

## 6. Discussion

Available evidence regarding the effects of propofol on cancer outcomes remains limited by the scarcity of high-quality randomized controlled trials and the predominance of retrospective studies, resulting in considerable heterogeneity in patient populations, perioperative management, and outcome measures. Although these limitations preclude definitive clinical conclusions, the available literature provides valuable mechanistic insights into tumor biology, perioperative immune responses, and potential translational pathways. Accordingly, this review was designed to integrate molecular mechanisms with perioperative and clinical evidence while explicitly distinguishing evidence according to its level of clinical validation. Specifically, mechanistic findings involving the PI3K/AKT/mTOR pathway, non-coding RNA regulation, epithelial–mesenchymal transition (EMT), apoptosis, and chemosensitivity are derived predominantly from experimental OSCC models and should therefore be interpreted as preclinical evidence. In contrast, conclusions regarding perioperative outcomes and long-term oncologic prognosis are based primarily on HNC-specific clinical studies, randomized controlled trials where available, and systematic reviews or meta-analyses. Evidence from mixed cancer populations or non-HNC surgical settings was included only when it provided direct mechanistic, immunological, or translational relevance and is clearly distinguished throughout the manuscript from HNC-specific clinical evidence to minimize inappropriate extrapolation.

Current evidence suggests that the role of propofol in HNC is considerably more complex than a simple tumor-suppressive or tumor-promoting model. However, the interpretation of this evidence requires careful consideration of study design and evidence level because mechanistic observations are derived primarily from experimental OSCC models, whereas clinical conclusions rely on a limited number of HNC-specific investigations and broader perioperative oncology studies. Although multiple experimental studies have demonstrated the anti-tumor properties of propofol, including the inhibition of proliferation, enhancement of apoptosis, and modulation of oncogenic signaling pathways, these findings remain difficult to translate into consistent clinical benefits [21,22,25,27,28,29,30,31,32,41]. Conversely, several studies have reported that propofol may enhance epithelial–mesenchymal transition (EMT), cell migration, and invasive behavior under specific experimental conditions [21,41]. These conflicting observations highlight a major unresolved question in perioperative oncology: Do the biological effects observed in preclinical systems accurately reflect clinically relevant perioperative exposure conditions [9,21,22]?

Another challenge in interpreting the current literature is the heterogeneity of both study design and evidence quality. Most mechanistic observations discussed in this review originate from experimental OSCC cell-line studies, whereas evidence regarding perioperative immune modulation and survival outcomes is largely derived from retrospective clinical studies, systematic reviews, meta-analyses, or mixed cancer cohorts. Consequently, biological plausibility should not be interpreted as equivalent to clinical efficacy, and mechanistic findings should be regarded primarily as hypothesis-generating rather than clinically validated evidence. Most of the mechanistic observations discussed in this review are derived from OSCC cell-line studies, whereas evidence regarding perioperative immune modulation and survival outcomes often originates from mixed cancer cohorts, retrospective analyses, or narrative reviews. Furthermore, HNC-specific prospective clinical data are limited and are largely confined to studies evaluating perioperative outcomes or comparisons between propofol-TIVA and INH. Therefore, differences in the study design, disease specificity, and level of evidence should be carefully considered when interpreting the translational relevance of the current findings.

One important limitation of the current literature is its heavy reliance on retrospective clinical studies and simplified in vitro models [7,8,9,20]. Several experimental studies have employed propofol concentrations exceeding clinically achievable perioperative plasma levels (approximately 2–6 μg/mL; 11–33 μM) and/or prolonged exposure durations (48–72 h), which may not accurately reflect perioperative pharmacokinetics. Consequently, the biological effects observed in vitro should be interpreted cautiously when extrapolating to clinical practice [21,22]. Furthermore, tumor biology in HNC is highly heterogeneous, and responses to anesthetic exposure may differ substantially according to tumor subtype, HPV status, metabolic phenotype, immune context, and treatment history [12,13,14,15,16]. These variables are rarely standardized across studies, which may partially explain the inconsistent findings reported in both mechanistic and clinical investigations [8,9,21].

The biological impact of propofol may differ substantially across HNC subtypes. HPV-positive OPSCC generally exhibits a more inflamed tumor microenvironment characterized by increased infiltration of CD8+ T cells, enhanced immune surveillance, and distinct molecular alterations compared with HPV-negative tumors. In contrast, OSCC and laryngeal cancers frequently arise in the setting of tobacco- and alcohol-associated carcinogenesis and often display different mutational profiles, stromal interactions, and immune-suppressive features [12,13]. Variations in HPV status, tumor location, immune-cell composition, angiogenic activity, and metabolic reprogramming may influence both perioperative host responses and tumor susceptibility to anesthetic-mediated biological effects [9,10,11]. Consequently, the oncologic impact of propofol-based TIVA may not be uniform across HNC subtypes, highlighting the importance of incorporating tumor-site and HPV-specific stratification into future clinical studies [12,13]. Because HPV-positive OPSCC is characterized by a T-cell-inflamed tumor microenvironment and greater dependence on anti-tumor immune surveillance [12,13], preservation of perioperative NK-cell and CD8^+^ T-cell function by propofol may have greater biological relevance in this subgroup than in HPV-negative, tobacco-associated HNC [9,11,18,19,25]. Conversely, HPV-negative, tobacco-associated HNCs generally exhibit a more immunosuppressive tumor microenvironment, in which the immunomodulatory effects of propofol may be less pronounced or biologically neutral [12,13]. Although this hypothesis remains speculative, it provides a plausible explanation for the heterogeneous clinical findings currently reported. Future mechanistic studies and prospective clinical investigations should therefore stratify experimental models and patient cohorts according to HPV status to determine whether baseline tumor immune context influences the biological and clinical effects of propofol.

In addition to propofol, other perioperative agents such as lidocaine have attracted attention because of their potential anti-inflammatory and anti-tumor properties [10,23,24]. Experimental studies have suggested that lidocaine suppresses tumor cell migration, reduces inflammatory cytokine release, and attenuates perioperative immune suppression [10,23]. Nevertheless, similar to propofol, current clinical evidence remains insufficient to establish definitive oncological benefits, and further comparative translational studies are required to clarify the relative contributions of different anesthetic and adjunctive strategies in perioperative cancer care [9,10,24].

Recent clinical evidence further supports the emerging concept that perioperative anesthetic and adjunctive strategies may influence the metabolic-inflammatory pathways associated with cancer progression. A recent clinical study investigating perioperative intravenous lidocaine during robotic-assisted radical prostatectomy demonstrated alterations in early biochemical and inflammatory parameters, suggesting a potential role for lidocaine in perioperative metabolic-inflammatory modulation [43]. These findings further support the hypothesis that perioperative anesthetic management extends beyond conventional analgesic and anesthetic functions and potentially influences perioperative immune and tumor-related biological responses. However, the translational relevance of these observations in HNC remains unclear and warrants further investigation.

Another unresolved issue is whether anesthetic exposure meaningfully alters long-term oncologic outcomes or whether perioperative factors such as surgical stress, systemic inflammation, opioid exposure, transfusion, and immune suppression exert a greater influence on tumor recurrence and metastasis [8,9,10,11,17]. The perioperative tumor microenvironment is shaped not only by direct tumor cell signaling but also by vascular, endothelial, and inflammatory alterations induced by surgical stress and perioperative interventions. Emerging evidence suggests that endothelial dysfunction, vascular remodeling, and angiogenic imbalance may influence immune cell trafficking, cytokine signaling, tissue hypoxia, and metastatic potential [10,11,18,24,25]. Clinicopathological observations of vascular remodeling disorders have further demonstrated that intimal thickening, smooth muscle cell proliferation, fragile collateral vessel formation, and microenvironmental instability can substantially alter local inflammatory and vascular responses [44]. These dynamic microenvironmental changes may contribute to the heterogeneous and context-dependent effects of propofol exposure during cancer surgery.

In this context, propofol may function less as a direct anti-cancer agent and more as a modulator of perioperative host responses [9,17]. Although the preservation of NK-cell activity and attenuation of inflammatory signaling have been proposed as potential mechanisms [18,19], direct evidence linking these immunological effects to improved survival outcomes in HNC remains limited [9,24,25].

Current evidence also raises broader conceptual questions regarding perioperative precision medicine. It remains unclear whether specific patient subgroups derive greater benefits from propofol-based anesthesia or whether biomarker-guided anesthetic strategies could be integrated into future perioperative cancer care [37,42]. Future studies should, therefore, move beyond descriptive comparisons between anesthetic techniques and instead adopt prospective, mechanism-driven approaches that integrate molecular profiling, immune monitoring, and clinically relevant exposure models [9,24,25,37,42].

Overall, the oncological significance of propofol in patients with HNC remains unclear. Rather than supporting a uniform biological effect, current evidence suggests that the influence of propofol is highly context-dependent and shaped by dynamic interactions among tumor biology, perioperative immunity, and host-related factors [9,17,21,24]. Addressing these unresolved questions requires multidisciplinary translational studies designed to bridge the gap between mechanistic observations and clinically meaningful outcomes.

Future HNC-specific clinical trials should move beyond simply comparing anesthetic techniques and instead adopt mechanism-driven translational study designs. In addition to standardized anesthetic protocols and biomarker-guided patient stratification (e.g., HPV status, immune phenotype, molecular subtype, and tumor site), future prospective studies should incorporate paired perioperative biospecimen collection to investigate the biological effects of propofol under clinically relevant conditions. Where feasible, paired tumor tissue obtained at diagnostic biopsy and surgical resection may be analyzed for EMT-related biomarkers, including SNAI1, E-cadherin, N-cadherin, and vimentin, using immunohistochemistry or transcriptomic approaches to evaluate whether propofol-based TIVA influences tumor-intrinsic molecular signaling. Serial peripheral blood sampling before anesthesia induction, immediately after surgery, and at 24 and 72 h postoperatively should also be considered to assess dynamic changes in circulating inflammatory cytokines (e.g., IL-6 and TNF-α), NK-cell activity, CD56^dim^/CD16^+^ NK-cell subsets, and T-cell populations. Primary clinical endpoints should include postoperative pulmonary complications and recovery quality, whereas secondary endpoints should evaluate recurrence-free survival, overall survival, perioperative immune responses, and biomarker-defined treatment effects. Such integrated translational trial designs would help determine whether perioperative molecular alterations translate into clinically meaningful oncologic outcomes.

**Figure 3:**
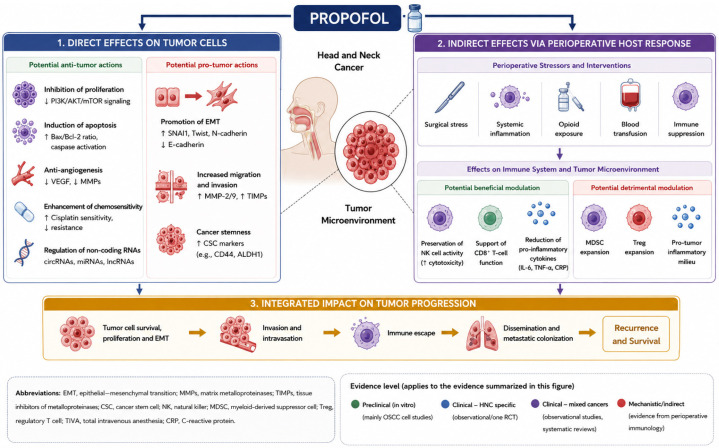
**Integrated framework of direct tumor-related and indirect host-mediated effects of propofol in head and neck cancer.** The figure summarizes currently available evidence regarding the potential influence of propofol on tumor biology and perioperative host responses. Direct effects are primarily derived from preclinical OSCC studies, whereas indirect effects are based on perioperative immunology and clinical investigations. The integrated impact on tumor progression represents a synthesis of existing evidence and should not be interpreted as proof of causality. Current HNC-specific clinical studies have not demonstrated a consistent improvement in overall survival or recurrence-free survival despite perioperative benefits associated with propofol-based anesthesia. The evidence incorporated into this framework reflects different evidence levels, including preclinical mechanistic studies, HNC-specific clinical investigations, and broader perioperative oncology literature, and should not be interpreted as representing equivalent levels of clinical validation. Blue and purple arrows indicate direct and indirect effects of propofol, respectively, whereas orange arrows indicate the integrated impact on tumor progression. This figure was generated with the assistance of ChatGPT (OpenAI, GPT-5.5) and subsequently curated by the authors to ensure accuracy in biological representation, terminology, and conceptual integration.

To better conceptualize the translational relevance of propofol in perioperative oncology, we proposed an integrated framework linking anesthetic exposure, tumor-intrinsic signaling pathways, perioperative immune modulation, and clinical outcomes in HNC (Figure 3). This framework highlights the context-dependent interactions between direct tumor cell effects, including the regulation of PI3K/AKT/mTOR signaling, EMT, apoptosis, and non-coding RNA networks, and indirect host-mediated mechanisms involving surgical stress, inflammatory responses, and perioperative immune dysfunction. The proposed model further emphasizes the potential role of biomarker-guided stratification and mechanism-informed anesthetic strategies in perioperative cancer care. Importantly, this framework is intended as a conceptual and hypothesis-generating model rather than a validated clinical algorithm, underscoring the need for prospective translational studies that integrate molecular profiling, immune monitoring, and clinically relevant exposure conditions. Throughout this review, HNC-specific clinical studies were interpreted separately from broader perioperative oncology literature. References from other cancer types were retained only when they provided mechanistic, immunological, or translational insights directly relevant to propofol-mediated effects or perioperative tumor biology. Consequently, conclusions regarding long-term oncologic outcomes were based primarily on HNC-specific clinical evidence, whereas findings from non-HNC studies were used only to provide biological context and hypothesis-generating support.

Importantly, most molecular mechanisms discussed in this review originate from OSCC experimental models, whereas conclusions regarding clinical outcomes are based primarily on HNC-specific clinical studies. Therefore, extrapolation of mechanistic observations to the broader HNC population should be made cautiously.

## 7. Conclusions

Propofol exerts dual and context-dependent effects on HNC, particularly OSCC, by modulating oncogenic signaling, apoptosis, EMT, and non-coding RNA networks. Although preclinical studies have suggested both tumor-suppressive and tumor-promoting activities, current HNC-specific clinical evidence does not demonstrate consistent improvements in OS, RFS, or overall long-term survival with propofol-based TIVA. Nevertheless, propofol-based TIVA has been associated with favorable perioperative outcomes, including reduced postoperative complications and improved recovery profiles. Nevertheless, perioperative advantages, including reduced postoperative complications and improved recovery, have been consistently observed. These discrepancies likely reflect tumor heterogeneity, perioperative confounders, and limitations inherent in the current evidence base. Future research should prioritize well-designed prospective trials and mechanism-based, biomarker-guided approaches to clarify the oncological relevance of propofol and refine anesthetic strategies for cancer surgery.

## Figures and Tables

**Table 1:** Classification and Hierarchy of Evidence Included in This Narrative Review.

Category	HNC-Specific Evidence	General Cancer Evidence	Predominant Evidence Level
Molecular mechanisms	OSCC molecular studies involving PI3K/AKT/mTOR signaling, apoptosis, and tumor progression [5,27,28,29,30]	Propofol-mediated regulation of oncogenic signaling pathways across multiple cancer types [5,21,22,31,32]	Preclinical
Non-coding RNA regulation	OSCC circ_0005623/miR-195-5p/HOXB7 and circ_0008898 pathways [27,28]	Non-coding RNA-mediated effects reported in various malignancies [21,32]	Preclinical
Chemoresistance	AREG-associated 5-FU resistance in OSCC [29]	Chemosensitization and modulation of treatment response in mixed cancer models [21,22]	Preclinical
Immune modulation	Limited HNC-specific evidence currently available [8,9]	NK-cell preservation, CD8^+^ T-cell activity, cytokine modulation (IL-6, TNF-α), and perioperative immune regulation [8,9,10,11,17,18,19,24,25]	Narrative reviews + Experimental
Perioperative outcomes	Reduced postoperative pulmonary complications (PPCs) following propofol-based TIVA in HNC surgery [33]	Improved perioperative recovery and reduced complications in various surgical populations [34,35]	Randomized clinical trial
Survival outcomes	HNC-specific studies showing no consistent improvement in OS or RFS [15,36]	Meta-analyses and mixed cancer cohorts reporting conflicting survival associations [4,7,20,37]	Meta-analysis + Retrospective studies
Evidence limitations	Limited HNC-specific prospective studies; predominance of OSCC-based mechanistic research [15,27,28,29,30,36,38]	Predominance of retrospective studies, mixed cancer cohorts, and heterogeneous perioperative conditions [7,8,9,20,24,37]	Overall evidence remains moderate-to-low

**Table 2:** Highest-Level Clinical Evidence Regarding Propofol and Cancer Outcome.

Study Design	Population	Primary Outcome	Principal Findings	Evidence Level	Reference
Meta-analysis of randomized controlled trials	Mixed cancer populations	OS	No significant improvement in long-term survival associated with propofol-based anesthesia	High	[4]
Systematic review and meta-analysis	Mixed cancer surgery	OS, RFS	Current evidence remains inconclusive because of study heterogeneity and risk of bias	High	[7]
Systematic review and meta-analysis	Mixed cancer populations	OS, RFS	Survival benefit reported in some retrospective studies but not consistently confirmed by higher-quality evidence	High	[20]
Randomized controlled trial	Patients undergoing HNC microvascular reconstruction	Postoperative pulmonary complications	Propofol-based TIVA significantly reduced postoperative pulmonary complications compared with inhalational anesthesia	High	[33]
Retrospective cohort study	HNSCC	OS	No significant association between propofol exposure and long-term survival	Moderate	[15]
Retrospective cohort study	OSCC	OS, RFS	No significant difference in OS or RFS between TIVA and inhalational anesthesia	Moderate	[36]

Abbreviations: HNC, head and neck cancer; TIVA, total intravenous anesthesia; OS, overall survival; RFS, recurrence-free survival; PPCs, postoperative pulmonary complications. Evidence hierarchy: Randomized clinical trials, systematic reviews, and meta-analyses were considered the highest level of clinical evidence, whereas retrospective cohort studies were classified as moderate-level evidence because of their susceptibility to selection bias and residual confounding.

**Table 3:** Preclinical Mechanisms of Propofol in Oral Squamous Cell Carcinoma Experimental Models.

Effect	Experimental Model	Molecular Pathway/ Target	Biological Effect	Ref.
Anti-tumor	OSCC cell line	circ_0005623/miR-195-5p/HOXB7	Downregulation of HOXB7 through circ_0005623-mediated sponging of miR-195-5p suppresses tumor progression.	[27]
Anti-tumor	OSCC cell line	circ_0008898	Inhibits proliferation, invasion, migration, and angiogenesis through modulation of downstream targets.	[28]
Anti-tumor	OSCC cell line	AREG	Downregulation reduces 5-fluorouracil (5-FU) resistance and induces apoptosis.	[29]
Anti-tumor	OSCC cell line	FoxO1/GAS5/miR-1297/GSK3β	Induces apoptosis and inhibits tumor growth through activation of this tumor-suppressive signaling cascade.	[38]
Pro-tumor	OSCC cell line	SNAI1/EMT	Upregulation of SNAI1 promotes epithelial–mesenchymal transition (EMT), cell migration, and invasion.	[41]

**Abbreviations:** OSCC, oral squamous cell carcinoma; EMT, epithelial–mesenchymal transition; circRNA, circular RNA; miRNA, microRNA; HOXB7, homeobox B7; AREG, amphiregulin; 5-FU, 5-fluorouracil; GAS5, growth arrest-specific transcript 5; GSK3β, glycogen synthase kinase 3 beta. All pathways summarized in this table are derived from experimental OSCC studies and are classified as preclinical mechanistic evidence. These findings provide biological insights into the potential actions of propofol but should not be interpreted as clinically validated mechanisms in patients with HNC.

## Data Availability

No new data were created or analyzed in this study. Data sharing is not applicable to this article.

## Abbreviations

The following abbreviations are used in this manuscript:

5-FU	5-fluorouracil
AREG	amphiregulin
EMT	epithelial–mesenchymal transition
GAS5	growth arrest-specific transcript 5
HNC	head and neck cancer
HNSCC	head and neck squamous cell carcinoma
HOXB7	homeobox B7
NK	natural killer
OS	overall survival
OSCC	oral squamous cell carcinoma
PPCs	postoperative pulmonary complications
RFS	recurrence-free survival
SNAI1	snail family transcriptional repressor 1
TIVA	total intravenous anesthesia
